# *In vivo* CaspaseTracker biosensor system for detecting anastasis and non-apoptotic caspase activity

**DOI:** 10.1038/srep09015

**Published:** 2015-03-11

**Authors:** Ho Lam Tang, Ho Man Tang, Ming Chiu Fung, J. Marie Hardwick

**Affiliations:** 1 W. Harry Feinstone Department of Molecular Microbiology and Immunology, Johns Hopkins University Bloomberg School of Public Health, Baltimore, MD 21205 USA; 2 School of Life Sciences and Center for Soybean Research of the State Key Laboratory of Agrobiotechnology, Chinese University of Hong Kong, Shatin, Hong Kong SAR, China

## Abstract

The discovery that mammalian cells can survive late-stage apoptosis challenges the general assumption that active caspases are markers of impending death. However, tools have not been available to track healthy cells that have experienced caspase activity at any time in the past. Therefore, to determine if cells in whole animals can undergo reversal of apoptosis, known as anastasis, we developed a dual color CaspaseTracker system for *Drosophila* to identify cells with ongoing or past caspase activity. Transient exposure of healthy females to environmental stresses such as cold shock or starvation activated the CaspaseTracker coincident with caspase activity and apoptotic morphologies in multiple cell types of developing egg chambers. Importantly, when stressed flies were returned to normal conditions, morphologically healthy egg chambers and new progeny flies were labeled by the biosensor, suggesting functional recovery from apoptotic caspase activation. In striking contrast to developing egg chambers, which lack basal caspase biosensor activation under normal conditions, many adult tissues of normal healthy flies exhibit robust caspase biosensor activity in a portion of cells, including neurons. The widespread persistence of CaspaseTracker-positivity implies that healthy cells utilize active caspases for non-apoptotic physiological functions during and after normal development.

Apoptotic caspases are proteases that cleave cellular substrates after specific aspartate residues to promote rapid self-destruction^1^,^2^,^3^. However, cells can survive beyond caspase activation either because caspases fail in their attempt to kill, or because caspases have non-apoptotic roles in healthy cells. The same caspases that orchestrate apoptosis have been implicated in diverse normal cell functions, such as regulation of neuronal activity^4^,^5^, learning and memory^6^,^7^,^8^,^9^, spermatid individualization^10^,^11^, suppression of necroptotic cell death^12^,^13^, and microRNA processing^14^. Dual life and death functions are not unique to caspases, but are shared by other classical apoptosis regulators, such as Bcl-2 family proteins, cytochrome *c*, and IAP (inhibitor of apoptosis) proteins^15^,^16^,^17^,^18^,^19^,^20^. Thus, evolution may have coordinately linked health and death functions to the same molecules to achieve appropriate elimination of dysfunctional cells. However, the extent of non-apoptotic caspase activity in whole animals is not known.

Surprisingly, normal mammalian cells and cancer cell lines can escape late stage apoptosis if the cell death stimulus is removed^21^,^22^,^23^. Partially dismantled cells with obvious apoptotic morphologies can repair themselves after passing important checkpoints, including cytochrome *c* release, caspase activation, DNA damage, nuclear condensation and fragmentation, and apoptotic body formation^21^,^22^,^23^. The term “anastasis” (Greek for “rising to life”) was adopted to describe this recovery from the brink of cell death^22^. Convalescent cells regain normal appearances and continue to survive and proliferate, including cells bearing chromosome rearrangements^22^,^23^. Thus, rare events of anastasis in animals potentially could promote tumorigenesis^21^,^22^,^23^,^24^. Conversely, beneficial anastasis in whole animals potentially could rescue apoptotic cardiomyocytes in from ischemic injury, or dying neurons from degeneration^22^,^25^. However, attempts to prove that anastasis indeed occurs *in vivo* have been hampered by the limited technologies for long-term tracking of cell fate following caspase activation in animals.

Successful biosensors have been developed to detect real-time caspase activity in cultured cells and animal tissues. The SCAT caspase biosensors (e.g. ECFP-DEVD-Venus) rapidly detect activate caspases using FRET^26^,^27^. The fluorescent Apoliner (mCD8-RFP-DQVD-NLS-eGFP) undergoes subcellular relocalization to the nucleus when its plasma membrane-tether is cleaved by caspases^28^, and ApoAlert pCaspase3-Sensor (NES-DEVD-YFP-NLS) relocalizes from the cytosol to the nucleus^22^,^23^,^29^. Immuno-detection of the caspase-cleaved form of CPV biosensors (e.g. CD8-PARP-Venus) was used to demonstrate caspase-dependent death of olfactory neurons during aging^30^,^31^. Currently available caspase biosensors do not permanently mark cells that have previously experienced transient caspase activity. To overcome this difficulty, we generated the dual color CaspaseTracker biosensor, which fluoresces red in cells with current or recent caspase activity and permanently fluoresces green to mark cells that have experienced caspase activity in the past, with or without on-going caspase activity. To track the fate of cells following caspase activation in live animals, we applied the CaspaseTracker biosensor to the *Drosophila melanogaster* model system.

## Results

### CaspaseTracker biosensor development

**Figure 1:**
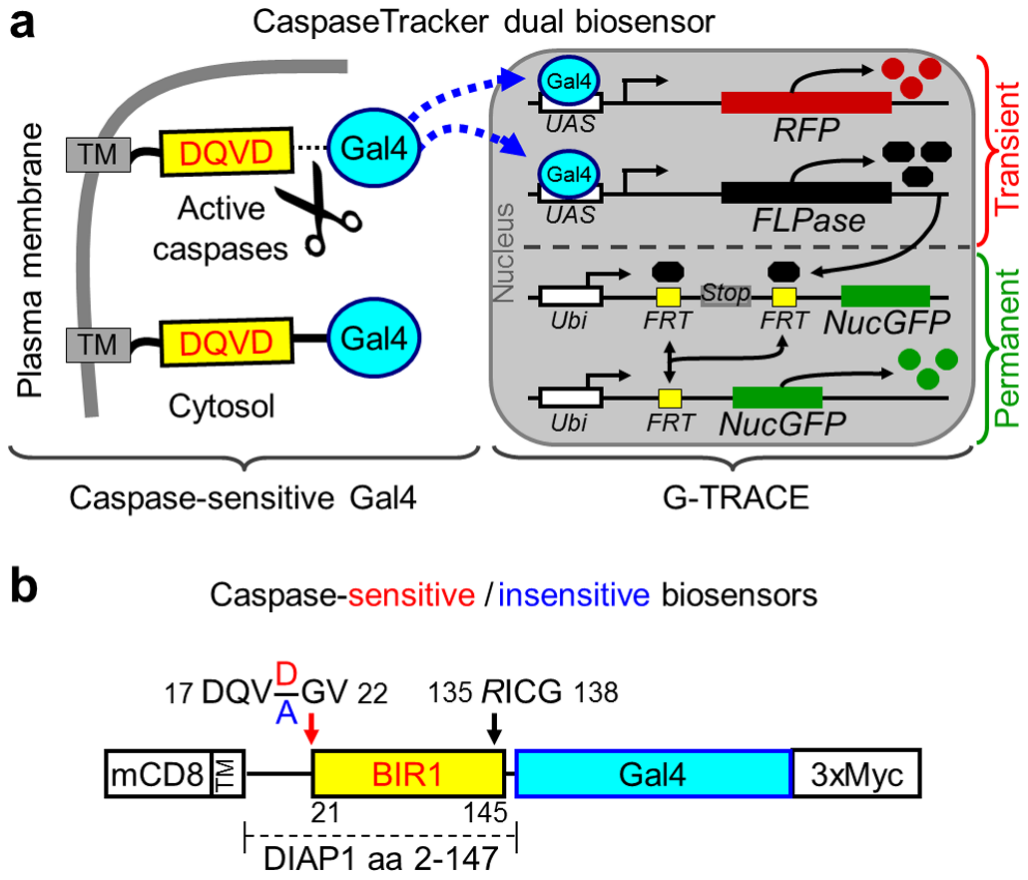
CaspaseTracker biosensor system. (a) Schematic of the CaspaseTracker biosensor system, which is composed of a plasma membrane anchor (mCD8), a caspase-sensitive natural substrate (derived from DIAP1) fused to the Gal4 transcription factor with a C-terminal 3x-myc tag that translocates to the nucleus upon caspase activation to induce the G-Trace system. (b) Schematic of caspase-sensitive (DQVD) and caspase-insensitive control (DQVA) biosensors with the indicated modifications (NN/GV and D135R)^33^,^34^ to prevent biosensor degradation and to prevent caspase inhibition by the biosensor.

The CaspaseTracker biosensor system is composed of two genetically encoded components, a caspase-activatable yeast transcription factor Gal4, and the G-TRACE fluorescent protein system^32^ (Fig. 1a). The caspase-activatable Gal4 is sequestered in the cytoplasm by a fragment of mammalian CD8 (mCD8) fused to a caspase-cleavable linker^28^. This 146-amino acid linker contains the natural caspase cleavage site DQVD near the BIR1 domain of *Drosophila* DIAP1 (Fig. 1b). Upon caspase activation, Gal4 is released from its plasma membrane tether, translocates to the nucleus and drives the expression of cytosolic red fluorescent protein (RFP) to indicate recent or on-going caspase activity, and simultaneously drives the expression of FLP recombinase leading to ubiquitous and permanent expression of nuclear-targeted GFP (NucGFP) for the life of the cell and its progeny (Fig. 1a). The DIAP1 linker contains two modifications to improve biosensor function, a 21NN/GV22 mutation to prevent degradation of Gal4 by the N-end rule upon caspase cleavage after Asp20^28^,^33^, and a D135R mutation known to abolish the drICE caspase inhibitory function of the sequence DICG in the BIR1 domain^34^ (Fig. 1b). Control biosensor flies were generated using the same biosensor with an additional engineered mutation of the obligatory Asp required for cleavage by caspases (DQVD to DQVA)^33^. For ubiquitous expression, the ubiquitin promoter was used to drive expression of the mCD8-DQVD/A-Gal4-myc CaspaseTracker biosensors. Both the caspase-activatable (DQVD) and control uncleavable (DQVA) biosensors appear to be expressed similarly based on immunostaining of whole mount transgenic *Drosophila* with anti-myc (Supplementary Fig. 1).

### CaspaseTracker detects apoptosis and anastasis

**Figure 2:**
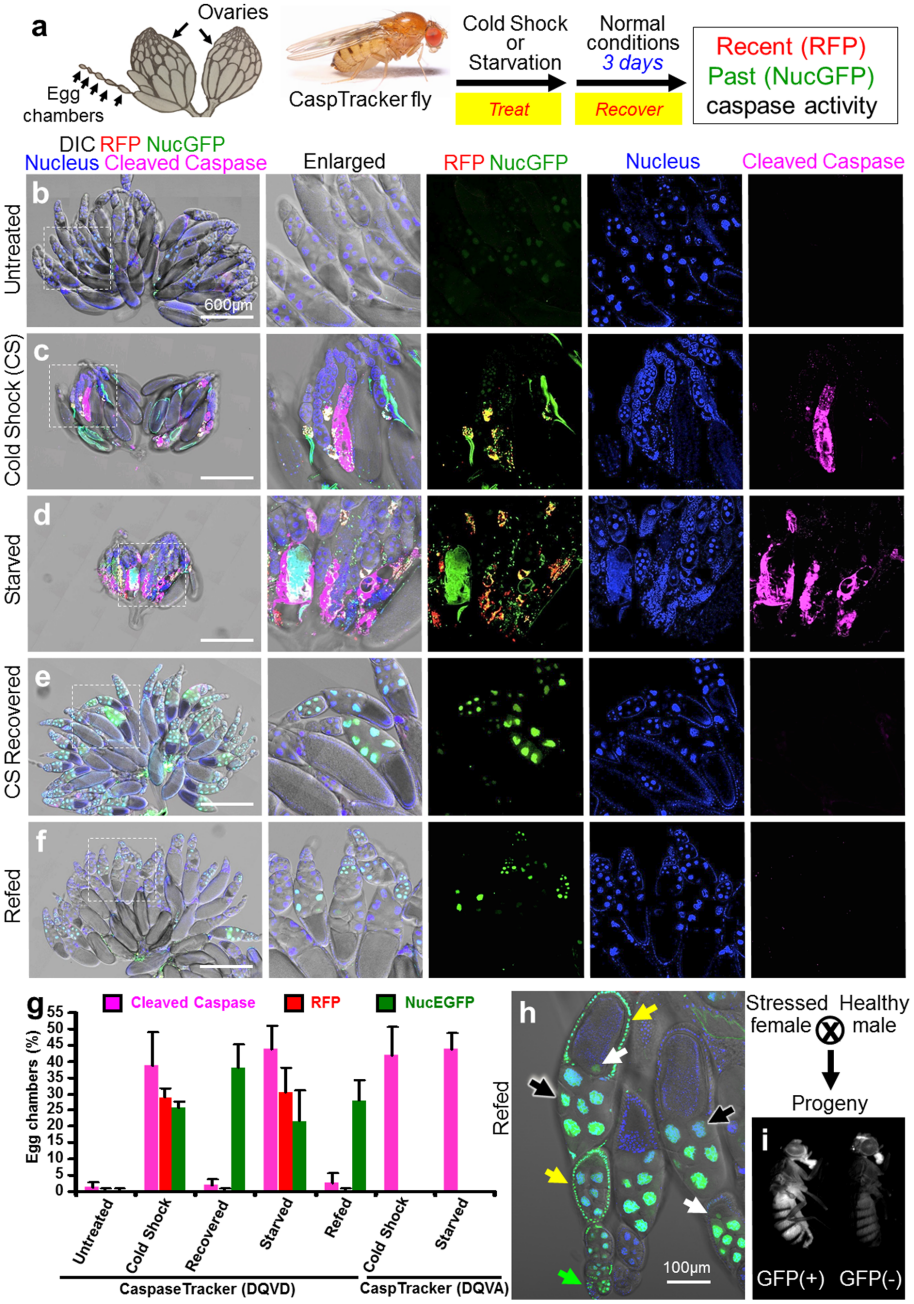
The CaspaseTracker System for detection of apoptosis and anastasis *in vivo*. (a) Schematic of *Drosophila* ovary, and flow chart for cold shock-, and protein starvation-induced cell death in 1-day-old flies, followed by 3-days recovery at normal condition. *Drosophila* ovary drawing is provided by Polan Santos; *Drosophila* image is provided by Darren Obbard. Used with permission (b) Egg chambers from the ovary of 6-day female CaspaseTracker flies fed with normal fly food for 6 days (untreated). (c) Caspase biosensor activity in egg chambers of CaspaseTracker *Drosophila* at 1 day after cold shock (−7°C, 1 hour, followed by 25°C for 24 hours) to induce apoptosis in egg chambers. (d) Caspase biosensor activity in egg chambers of CaspaseTracker *Drosophila* fed 3 days with 8% sucrose in 1% agar (starved) to induce apoptosis in egg chambers. (e) Like panel *c* except flies were then switched to normal conditions for 3 days after cold shock (CS recovered). (f) Like panel *d* except flies were switched to normal yeast-based fly food for 3 days after starvation (refed). Panel at left most is merged confocal image of RFP, NucGFP, nuclei, cleaved-caspase immuno-staining and DIC for overview of egg chambers at the ovaries; middle left panel is enlarged view of the dotted box at the left most panel; middle, middle right, and right most panels display biosensor RFP and NucGFP, nucleus, and cleaved caspase, respectively. (g) Quantification of RFP and NucGFP expression in egg chambers of CaspaseTracker (DQVD) flies before and after apoptosis induction. Caspase insensitive CaspaseTracker (DQVA) files serve as controls. Data presented are from 3 different batches of flies (n = 20), counting 100 egg chambers from each batch per condition. Error bars denote SD. (h) Confocal image of egg chambers recovered 3 days after starvation. Nuclear GFP in nurse cells (black arrows), oocytes (white arrows) and follicle cells (yellow arrows) of egg chambers, and in the germarium (green arrow). (i) GFP and non-GFP expressing progeny from starved and refed CaspaseTracker (DQVD) female flies.

Environmental insults such as cold shock and protein starvation cause physiological stress that triggers cell death including death by apoptosis in *Drosophila*^35^,^36^,^37^. Cold shock can cause massive cellular damage such as loss of cell membrane selective permeability^38^,^39^. Cell death induced by starvation during *Drosophila* oogenesis is due in part to caspases, apparently to match female fertility to environmental resources^40^,^41^. CaspaseTracker (DQVD) biosensor flies were analyzed the day after a cold shock (1 hour at −7°C) and after 3 days of protein starvation (8% sucrose in 1% agar). Egg chambers in the ovaries of only the treated flies developed both cytoplasmic red (recent/ongoing) and nuclear green (permanent) biosensor activity (Fig. 2a–d). Biosensor activation was accompanied by evidence of apoptosis, including nuclear condensation and immunoreactivity for cleaved effector caspases, a marker of activated caspases in mammals and *Drosophila*^36^,^42^,^43^ (Fig. 2c and d, and Supplementary Figs. 2 and 3). In contrast, mutation of the caspase cleavage site in the control caspase-insensitive (DQVA) biosensor abolished CaspaseTracker activity, indicating caspase-specific cleavage after the known Asp cleavage site in the DQVD biosensor. This difference was not explained by the lack of cell death in DQVA flies, as egg chambers from both DQVD and DQVA biosensor flies exhibited similar morphological changes and cleaved/active caspase immunoreactivity (Supplementary Figs. 2 and 3).

Our previous studies using a transient caspase biosensor (NES-DEVD-YFP-NLS) demonstrated that cultured mammalian cells can reverse apoptotic cell death after caspase activation if the apoptotic stimulus is removed^22^,^23^. To test if this anastasis phenomenon can also occur *in vivo*, CaspaseTracker flies from the same cold-shocked and protein-starved cohorts were allowed to recover 3 days in normal conditions (Fig. 2a). Strikingly, egg chambers after recovery displayed GFP (past), but not RFP (recent/ongoing) caspase biosensor activity nor staining of cleaved caspase, indicating that egg chambers can reverse cell death pathways (Fig. 2e-g). Again, CaspaseTracker specifically detects caspase activation based on the absence of biosensor activity in treated control caspase-uncleavable CaspaseTracker (DQVA) (Fig. 2g, and Supplementary Figs. 2 and 3).

Multiple cell types within egg chambers appear capable of reversing apoptosis and repairing the damage from caspases activated by cold shock or starvation, including both somatic (follicle) cells and germline cells (nurse cells and oocytes) (Fig. 2h, and Supplementary Fig. S4). Interestingly, the GFP (past) caspase biosensor simultaneously labeled cells in the germarium, where stem cells reside, along with its associated egg chambers in the same ovariole chain (Fig. 2h, and Supplementary Fig. S4). Thus, these biosensor-positive egg chambers may have been derived from stem cells that underwent anastasis. Importantly, the emergence of GFP-positive progeny flies from stressed female flies indicates that GFP-positive egg chambers are competent to produce offspring (Fig. 2i). Although green progeny flies were not further studied, they appeared normal and healthy. The long-term survival of cells following cold shock- and starvation-induced apoptotic morphologies and concomitant caspase activity provides the first evidence that apoptosis is reversible in a live animal, and further demonstrates the utility of CaspaseTracker as an *in vivo* biosensor of anastasis.

### Widespread non-apoptotic caspase activity detected by CaspaseTracker

**Figure 3:**
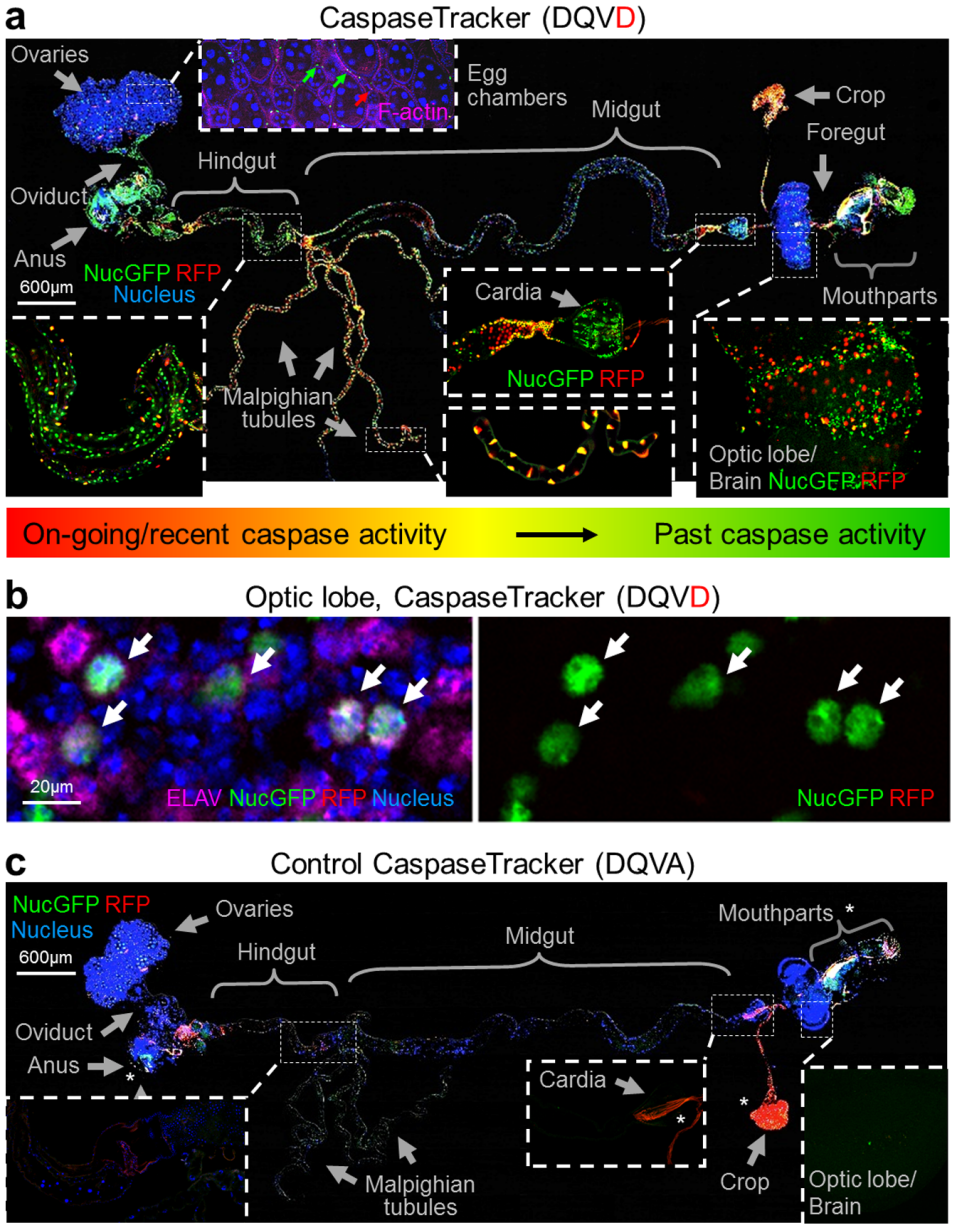
Physiological caspase activity during development (a) Merged confocal image of RFP and NucGFP biosensor, Hoechst for nuclei and DIC of whole mount dissection of newly eclosed day 0 caspase-sensitive CaspaseTracker (DQVD) fly raised at 18°C. Phalloidin for F-actin (pink) is shown only at the sub-panel for egg chambers. (b) NucGFP colocalizes with pan neuronal nuclear immunostaining for ELAV in the optic lobe of CaspaseTracker (DQVD) fly brain. Arrows indicate the co-localized signals of NucGFP and ELAV. (c) Merged confocal image of whole mount dissection of newly eclosed caspase-insensitive CaspaseTracker (DQVA) fly raised at 18°C, imaged with the same condition as panel *a*. *Autofluorescent regions.

An initially unexpected outcome of this study was the extensive basal CaspaseTracker (DQVD) biosensor activity observed in many tissues of newly eclosed day 0 flies, apparently due to normal developmental caspase activation (Fig. 3a). Although *Drosophila* egg chambers lack spontaneous caspase activity in the absence of a death stimulus, the adjacent muscle cells in the ovary were biosensor-positive in healthy untreated flies (Fig. 3a, and Supplementary Fig. 5). Interestingly, both on-going/recent (red) and past (green) caspase biosensor activities are particularly prominent in the brain and optic lobes, but are also abundant throughout the gut (foregut, midgut and hindgut) and Malpighian tubules, as well as the cardia and other tissues in female and male flies (Fig. 3a, and Supplementary Figs. 6 and 7). Non-apoptotic functions of caspases are better studied in neurons of both mammals and *Drosophila*^4^,^5^,^6^,^7^,^8^,^9^. To determine if neurons display non-apoptotic caspase activity detected by our CaspaseTracker biosensor, adult optic lobes were immunostained with the pan-neuronal antibody to ELAV, a *Drosophila* neuron-specific protein^44^. The GFP (past) biosensor prominently co-localized with the ELAV signal (Fig. 3b), consistent with non-cell death functions of caspases in neurons.

GFP-only cells detected in newly eclosed flies may include stem cells and their progeny that survived metamorphosis. In contrast to most tissues, the crop, oviduct, and male ejaculatory duct contain predominantly GFP (past) but not RFP (ongoing/recent) caspase biosensor activity, further suggesting that these cells likely utilize caspases for unknown non-apoptotic developmental functions (Fig. 3a, Supplementary Figs. 6 and 7). As expected, non-transgenic W118 flies lacked both GFP and RFP biosensor activity (Fig. 3c, and Supplementary Fig. 8). The well-known autofluorescence from the cuticle of mouthparts, labellum, pseudotracheae, and of ingested food such as yeast in the gut and crop are similar in biosensor and non-transgenic flies^45^,^46^.

### Spontaneous activation of caspases in adults

**Figure 4:**
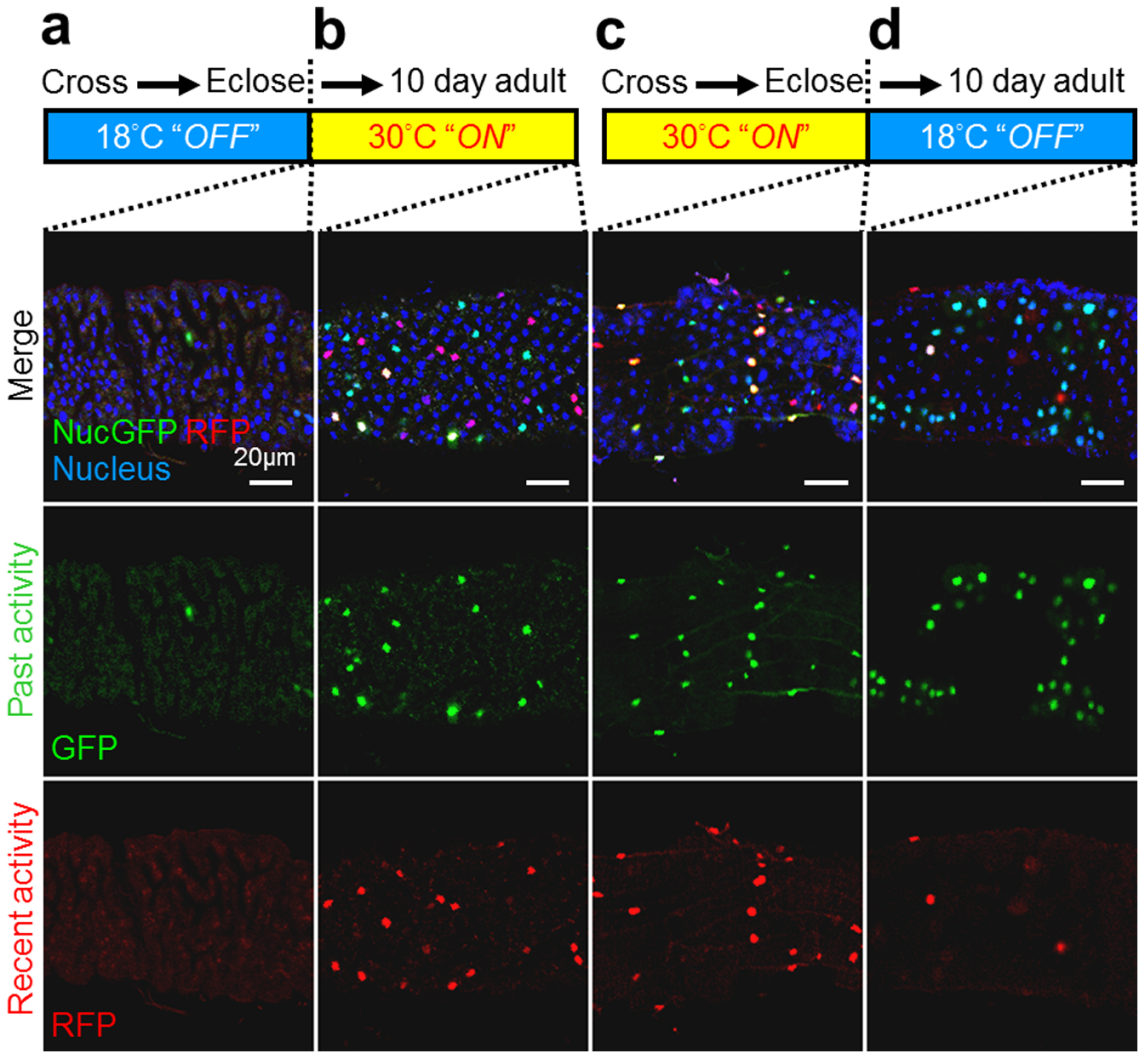
Post developmental caspase activation, and persistence in the adult of cells that experienced developmental caspase activity at *Drosophila* anterior midgut. Biosensor fluorescence of anterior midgut of (a) Newly eclosed Gal80^ts^–temperature sensitive(ts) CaspaseTracker flies raised at 18°C. The thermosensitive Gal80 (Gal80^ts^) conditionally represses Gal4 at 18°C; biosensor is functional “Off” during development. (b) Newly eclosed 18°C raised (ts) adult CaspaseTracker flies were then shifted to 30°C for 10 days. Gal80^ts^ cannot repress Gal4 at 30°C; biosensor is functional “On” at the 10-day period. (c) Newly eclosed tsCaspaseTracker flies raised at 30°C; biosensor is functional “On” at the development. (d) Newly eclosed 30°C raised (ts) adult CaspaseTracker flies were shifted to 18°C for 10 days. Gal80^ts^ represses Gal4 at 18°C; biosensor is functional “Off” at the 10-day period.

Like human gut tissue, the *Drosophila* gut also undergoes considerable cell turnover^47^, potentially consistent with the RFP (ongoing/recent) caspase biosensor activity observed throughout the gut, but particularly in regions of the hindgut (Fig. 3a). To determine if cell turnover in the *Drosophila* midgut also occurs in adults, we generated new biosensor flies with a Gal80^ts^ temperature-sensitive (ts) biosensor (Supplemental Fig. 9). Gal80^ts^ binds and suppresses Gal4 activity at 18°C^48^, turning off the biosensor, consistent with only rare biosensor-positive cells in tsCaspaseTracker flies raised at 18°C (Fig. 4a). However, the inhibitory effect of Gal80^ts^ was abolished at 30°C^48^, which turns on the biosensor. Therefore, shifting newly eclosed adult flies from 18°C to 30°C resulted in both GFP and RFP biosensor-positive cells 10 days later in midguts (Fig. 4b) and elsewhere. The presence of RFP biosensor activity at 10 days strongly indicates that caspases were activated in adults.

### Long-term cell survival after caspase activation

If indeed our CaspaseTracker biosensor marks healthy durable cells, and does not simply mark cells destined to die within a few days, then GFP-only cells should persist long-term. Newly eclosed temperature-sensitive tsCaspaseTracker flies raised with the biosensor turned on at 30°C during development exhibited abundant RFP and GFP biosensor activity as expected (Fig. 4c). To test the long-term survival of cells after caspase activation, newly eclosed adult flies were shifted to 18°C to turn off the biosensor, confirmed by the low RFP (ongoing/recent) biosensor activity 10 days later (Fig. 4d). Interestingly, the GFP (past) biosensor was readily detected at 10 days in midguts (Fig. 4d) and elsewhere. Thus, cells that experienced developmental caspase activity may survive for at least a week into the adult stage.

## Discussion

We have generated a powerful caspase biosensor to distinguish and track cells that have undergone anastasis in live animals. Caspase activation can cause cell demolition and death in less than 30 minutes^26^,^49^,^50^. Therefore, apoptosis is often considered to be an irreversible cell suicide process after caspase activation^2^,^24^,^51^,^52^,^53^,^54^,^55^. Using a mammalian cell caspase biosensor, we previously showed that cultured cells can recover from late-stage apoptosis^22^,^23^. Those studies in cultured cells have now been extended to whole animals using the new *Drosophila* CaspaseTracker biosensor as described here, We found that egg chambers of animals exposed to transient environmental stresses can recover from caspase activity and complete development to the adult stage. This biosensor marks convalescent cells with normal morphology, suggesting that biosensor-positive cells were dying cells that had recovered from activated caspases, rather than replacement cells acquired through division of unaffected cells. We cannot however, eliminate the possibility that some of the observed caspase activity in treated animals instead contributes to cell recovery. However, because biosensor activity can occur in the same egg chambers that also contain active caspase immunoreactivity and apoptotic morphologies, it appears that the purpose for at least a portion of the observed caspase biosensor activity was to cause cell death. Furthermore, caspases have established roles in promoting stress-induced cell death in egg chambers^2^,^35^,^36^,^37^,^54^. The portion of caspase activity devoted to cell destruction versus cell repair is difficult to evaluate, in part because each method used to observe caspase activity detects a different phase of the death process that are understandably not found in the same cell at the same time. For example, anti-active caspase immunoreactivity is expected several hours prior to expression of the RFP biosensor, which detects recent but not immediate caspase activity, while the GFP biosensor that detects prior caspase activity takes longer to be activated as it involves caspase activated Gal4 driven FLP-FRT recombination.

The second major finding with the CaspaseTracker biosensor system is the abundance of healthy biosensor-positive cells throughout the tissues of healthy flies reared under optimized conditions. This suggests widespread use of caspases for yet undefined non-death functions in normal healthy cells. However, the same caveat for interpreting the death function of caspases, as discussed above, also applies to the interpretation of normal day-job functions of caspases. That is, some portion of the caspase activity detected in normal healthy flies may reflect the normal rate of cell death, which is estimated to be tens of billions per day in humans^56^,^57^,^58^. However, based on the apparently normal morphology of cells expressing the biosensor, including the RFP biosensor for recent/ongoing caspase activity, it appears highly likely that CaspaseTracker is capable of detecting caspase activity intended for normal, not death-related functions in healthy cells. Widespread basal biosensor activity in the CaspaseTracker fly suggests that normal basal caspase activity is not limited to the examples reported thus far.

This *in vivo* CaspaseTracker biosensor system facilitates pursuit of the physiological role of anastasis, a potential natural healing mechanism to rescue cells from death threats induced by stress. Although the mechanisms of anastasis are not yet known, autophagy has been suggested to promote cell recovery from death, though without distinguishing long-term recovery from cell death inhibition^59^. Strategies to promote anastasis could potentially be beneficial in treating heart failure and neurodegeneration, while inhibiting anastasis during cancer therapy could potentially suppress cancer recurrence^21^,^22^,^23^,^24^,^25^. DNA damage is a hallmark of apoptosis^51^,^60^, and we observed a measurable increase in chromosome abnormalities in primary mammalian cells and cell lines that have undergone anastasis^22^,^23^. Therefore, anastasis in normal cells might represent a mechanism of tumorigenesis, for example in tissues repeatedly exposed to apoptosis-inducing agents^61^. Alternatively, anastasis in cancer cells could potentially promote the emergence of drug-resistant tumors^62^,^63^. Interestingly, our results with the *Drosophila* CaspaseTracker system indicate that anastasis can occur in germ cells after transient exposure of flies to apoptosis-inducing environmental stresses. If damaged germ cells can produce successful progeny, anastasis could promote genetic diversity to accelerate natural selection after mutagenic environmental insults^22^, as previously proposed in plants, yeast and bacteria^64^,^65^,^66^,^67^.

The CaspaseTracker system detects cells with non-apoptotic caspase activity in normal physiological conditions both during development and in adults. Intriguingly, large numbers of healthy biosensor-positive cells that have previously experienced caspase activity can persist in many tissues of optimally reared flies, consistent with widespread non-apoptotic functions of caspases in live animals^6^,^7^,^8^,^9^,^10^,^11^,^12^,^13^,^14^,^15^,^18^,^19^. Restricted levels of caspase activity in healthy neurons may dismantle synaptic endings, potentially by apoptosis-like processes^6^,^7^,^8^,^9^. However, molecular mechanisms entirely distinct from non-lethal, apoptosis-like caspase activity may be prevalent based on the widespread expression of the caspase biosensor in adult flies. The substrates of healthy caspases may or may not overlap with the substrates cleaved during cell death. Whether the concentration of active caspases is the critical determinant of life versus death, or if altered substrate preferences are also important, *Drosophila* is an important model for understanding this basic biology^68^,^69^,^70^. This biosensor will help dissect the molecular mechanisms and therapeutic implications of anastasis and non-apoptotic caspase activity.

## Methods

### Molecular cloning and generation of CaspaseTracker system

DNA encoding fragments of plasma membrane targeting mammalian CD8 and a cleavage site (DQVD) containing a fragment of the BIR1 domain (NN/GV) provided by Jean-Paul Vincent (Institute of Cancer Research, Fulham Road, London)^28^ were amplified by polymerase chain reaction (PCR) to obtain the CD8-BIR1 fragment with a Kozak sequence (GCCACC) at the 5’ start codon (ATG), and subsequently modified by site-directed mutagenesis (DICG/RICG). Gal4 DNA was amplified by PCR. Synthetic 3xMyc DNA with a 3’ stop codon (TAA) was produced by gBlocks (Integrated DNA Technologies). A four-component assembly In-Fusion (Clontech, 638910) was used to assemble CD8-BIR1, Gal4, and 3xMyc DNA fragments into the EcoRI - XbaI sites of the pUWR vector with a poly-ubiquitin promoter and Hsp27 terminator (*Drosophila* Genomics Resource Center, stock number 1281). DQVD was changed to DQVA for the control biosensor. Transgenic flies were generated at BestGene, Inc., and caspase sensitive and insensitive Gal4 flies were crossed with G-TRACE flies (Bloomington *Drosophila* Stock Center, stock number 28280) to produce CaspaseTracker flies. All 7 founder lines of DQVD biosensor flies expressed similar basal biosensor activity, while none of the 10 DQVA lines expressed biosensor activity. Gal80^ts^ flies (Bloomington *Drosophila* Stock Center, stock number 27018) were crossed to generate CaspaseTracker Gal80^ts^ files.

### Induction of apoptosis *in vivo*

Shortly after emergence, adult flies were fed with fly food (Nutri-Fly™ Bloomington Formulation, cat. no. 66–112) with yeast paste for 1 day. For cold shock induction, the flies were placed at −7°C for 1 hour. For protein starvation, flies were fed with 8% sucrose in 1% agar for 3 days. To allow reversal of apoptosis to occur, the induced fed flies were then re-fed with normal fly food (Nutri-Fly™ Bloomington Formulation, cat. no. 66–112) with yeast paste for 3 days. All flies were kept at 18°C except where indicated.

### Immunostaining and confocal microscopy

After dissection, fly tissues were fixed with 3.8% paraformaldehyde in PBS (v/v) for 20 minutes at room temperature and washed 3 times with PBS. Fixed tissues were permeabilized with 0.03% Triton X-100 in PBS, (PBST) (v/v) for 1 hour, and stained with 250 ng/ml Hoechst 33342 (Molecular Probes, cat. no. H1399) and 0.3 μM F-actin (filamentous actin) stain Alexa Fluor 633 Phalloidin (Molecular Probes, cat. no. A22284) in PBST for 1 hour at room temperature, and washed 3 times with PBST every 5 minutes. To detect caspase activation by immunostaining, egg chambers were first incubated with the cleaved caspase-3 (Asp175) antibody (Cell signaling technology, 9661) diluted 1:200 in PBST with 1% bovine serum albumin (BSA) (v/v) at 4°C overnight, and then Alexa Fluor 633 Goat Anti-Rabbit IgG antibody (Molecular Probes, A-21070) diluted 1:200 in PBST with 1% BSA (v/v) at room temperature for 2 hours and washed 3 times with PBST after incubation of antibody. The stained tissues were mounted on glass coverslips with Vectashield mounting medium (Vector laboratories, H-1000). Images were captured with Zeiss LSM 780 confocal inverted microscope using a 20x, NA 0.8 Plan-Apochromat objective, and were analyzed using Zen 2013 or AxioVision 4.2 software (Carl Zeiss). Differential interference contrast (DIC) microscopy was used to image the morphology of tissues.

### Imaging of GFP expressing progeny from CaspaseTracker female flies

GFP signal of flies was captured by SMZ1500 fluorescent zoom stereomicroscope (Nikon) with a CoolSNAP EZ Monochrome camera (Photometrics).

## Author Contributions

H.L.T., H.M.T., M.C.F. and J.M.H. conceived the ideas, directed the work, analyzed data, and wrote the paper. H.L.T. and H.M.T. designed and performed all experiments with input from all co-authors.

## Supplementary Material

Supplementary Information Supplementary Information
