# Supplementary material for: In vivo CaspaseTracker biosensor system for detecting anastasis and non-apoptotic caspase activity
Source: Sci Rep. 2015 Mar 11;5:9015. doi: 10.1038/srep09015 (PMC4355673; doi:10.1038/srep09015)
Supplement: Supplementary Information [file srep09015-s1.pdf]

Supplementary Information

In vivo CaspaseTracker biosensor system for detecting anastasis and non-apoptotic caspase activity

Ho Lam Tang[1,2*], Ho Man Tang[1], Ming Chiu Fung[2*] and J. Marie Hardwick[1*]

[1]W. Harry Feinstone Department of Molecular Microbiology and Immunology, Johns Hopkins University Bloomberg School of Public Health, Baltimore, MD 21205 USA

[2]School of Life Sciences, Chinese University of Hong Kong, Shatin, Hong Kong SAR, China

* Manuscript correspondences

Ho Lam Tang, Ph.D, Johns Hopkins University, Bloomberg School of Public Health, 615 N Wolfe St., MMI-BSPH, Baltimore, MD 21205 USA, Ph 443-813-1947; Fx 410-955-0105, holamtang@jhmi.edu
(Communication for manuscript submission)

Ming Chiu Fung, Ph.D. School of Life Sciences, Chinese University of Hong Kong, Shatin, Hong Kong SAR, China, Ph (852) 3943 6147; Fx (852) 2603-5646, mingchiufung@cuhk.edu.hk

J. Marie Hardwick, Ph.D, Johns Hopkins University, Bloomberg School of Public Health, 615 N Wolfe St., MMI-BSPH, Baltimore, MD 21205 USA, Ph 410-955-2716; Fx 410-955-0105, hardwick@jhu.edu

Contents:

1. Supplementary Figure Legends 1 to 9

2. Supplementary Figures 1 to 9

Supplementary Figure Legends

Supplementary Figure S1

Expression of caspase-activatable Gal4 biosensor *in vivo*.

DIC and fluorescence confocal microscopy images of transgenic female flies expressing the caspase-activatable mCD8-DQVD-Gal4-Myc CaspaseTracker, and the control mCD8-DQVA-Gal4-Myc version of the same with a single amino acid change rendering the biosensor caspase uncleavable. Indicated fly tissues were immunostained with anti-Myc. Scale bars 300 μm.

Supplementary Figure S2

Caspase-insensitive control CaspaseTracker (DQVA) biosensor cannot be activated by cold shock in *Drosophila* egg chambers despite comparable caspase activation.

Confocal images of egg chambers from caspase-sensitive CaspaseTracker (DQVD) and control (DQVA) biosensor flies at 1 day after cold shock (-7 ^{0}C for 1 hour) immunostained with antibody against cleaved/active caspase-3 (pink) and with Hoechst to mark nuclei (blue). Egg chambers that display caspase staining also exhibit nuclear condensation (arrows) characteristic of apoptosis (examples marked with dashed ovals). Note that both DQVD and control biosensors flies have similar non-specific green autofluorescence. Scale bar, 100 μm.

Supplementary Figure S3

Caspase-insensitive control CaspaseTracker (DQVA) biosensor cannot be activated by protein starvation in *Drosophila* egg chambers despite comparable caspase activation.

Confocal images of egg chambers from caspase-sensitive CaspaseTracker (DQVD) and control (DQVA) biosensor flies that were fed with 8% sucrose in 1% agar without protein for 3 days. Immunostaining with antibody against cleaved/active caspase-3 (pink) and with Hoechst to mark nuclei (blue). Egg chambers that display caspase staining also exhibit nuclear condensation (arrows) characteristic of apoptosis (examples marked with dashed ovals). Scale bar, 100 μm.

Supplementary Figure S4

Anastasis of germ cells, somatic cells and cells at germarium after recovery from cold shock.

Confocal images of egg chambers from CaspaseTracker (DQVD) flies after 3 days recovery from cold shock. Nuclear GFP in nurse cells (black arrows), oocytes (white arrows) and follicle cells (yellow arrow) of egg chambers, and cells in the germarium that resemble stem cells (green arrow). Scale bar, 100 μm.

Supplementary Figure S5

Basal RFP (recent) and GFP (past) CaspaseTracker (DQVD) biosensor activity in the ovary is primarily limited to muscle sheets.

Image of egg chambers enlarged from Fig. 3a. Phalloidin stains muscle F-actin; Hoechst stains nuclei. White arrows indicate same cells across images. Yellow arrows indicate autofluorescence from oocytes of egg chambers at red channel. Scale bar, 50 µm.

Supplementary Figure S6

Survival of developmental caspase activity

Enlargements of DIC and fluorescence confocal images of GFP (past) and RFP (recent) CaspaseTracker (DQVD) activity and Hoechst-stained nuclei in the brain, cardia, crop, midgut, Malpighian tubules and oviducts from Fig. 2a. Scale bar, 100 µm

Supplementary Figure S7

Physiological caspase activity in adult male *Drosophila*

Merged confocal images of RFP (recent), NucGFP (past) CaspaseTracker (DQVD) activity and Hoechst-stained nuclei of whole mount dissections of newly eclosed caspase-sensitive CaspaseTracker (DQVD) male flies raised under the same conditions before dissection as flies at figure 2. Scale bar, 600 µm.

Supplementary Figure S8.

Whole mounts of non-transgenic flies (W118).

Representative confocal images of whole mount dissected non-transgenic flies (W118), raised under the same conditions before dissection as flies at figure 2. Scale bar, 600 µm.

Supplementary Figure S9

Schematic of Gal80^{ts}-based temperature-dependent on-off switch of CaspaseTracker biosensor system

(a) Gal80^{ts} binds and inhibits Gal4 at 18^{0}C so that CaspaseTracker system is off.

(b) The inhibitory effects of Gal80^{ts} are abolished at 30^{0}C to turn on the CaspaseTracker (DQVD) biosensor.

Tang et al. Supplementary Figure 1

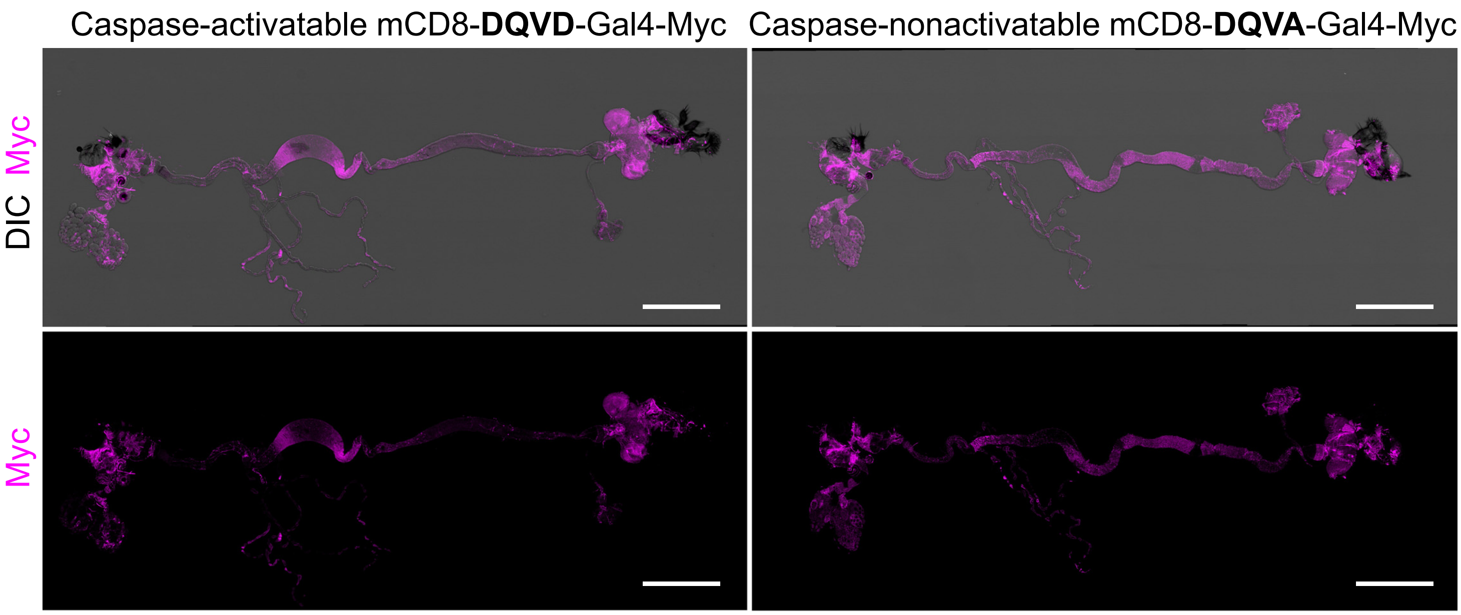

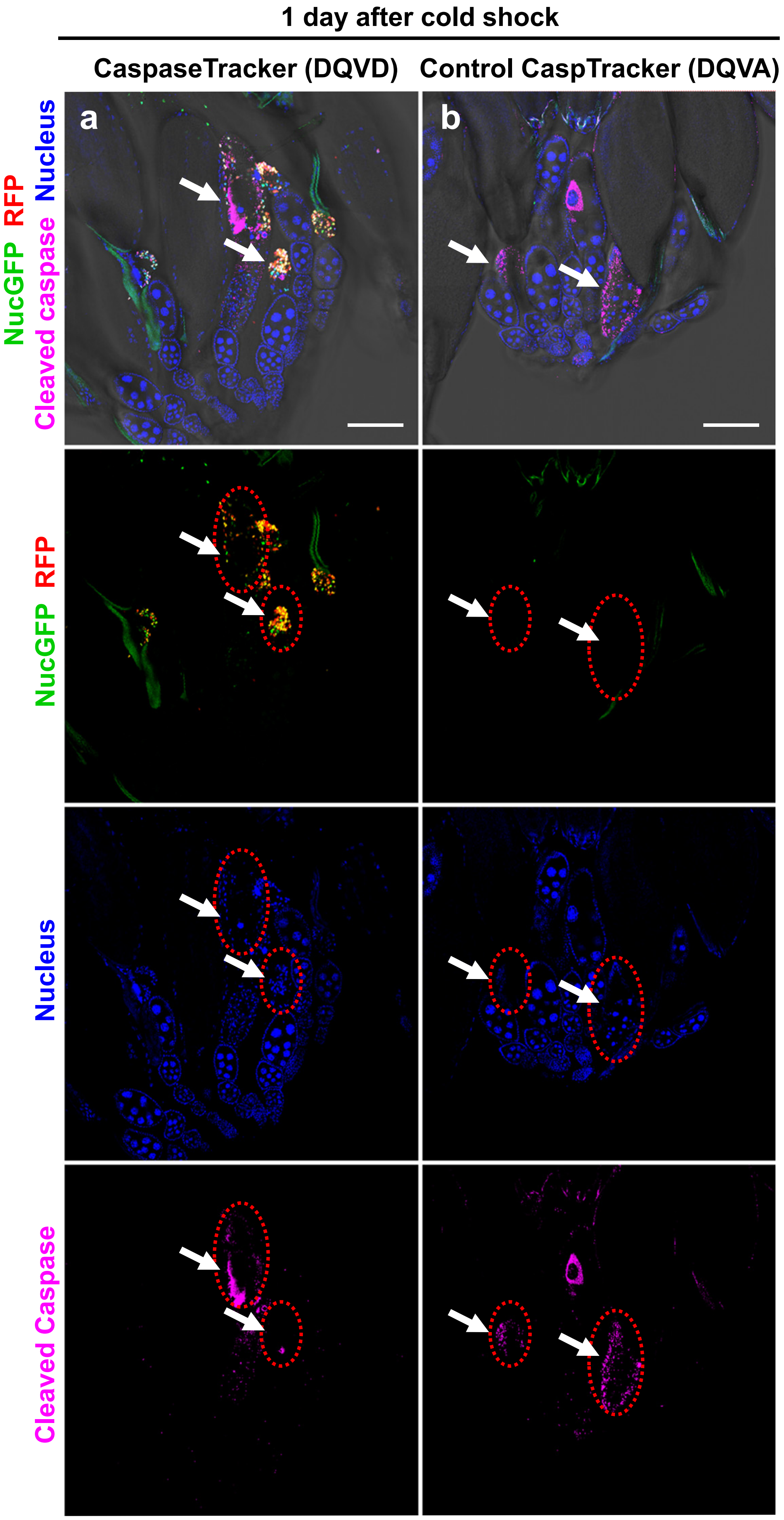
1 day after cold shock
CaspaseTracker (DQVD)
Control CaspTracker (DQVA)
a
b
NucGFP RFP Cleaved caspase Nucleus
NucGFP RFP
Nucleus
Cleaved Caspase

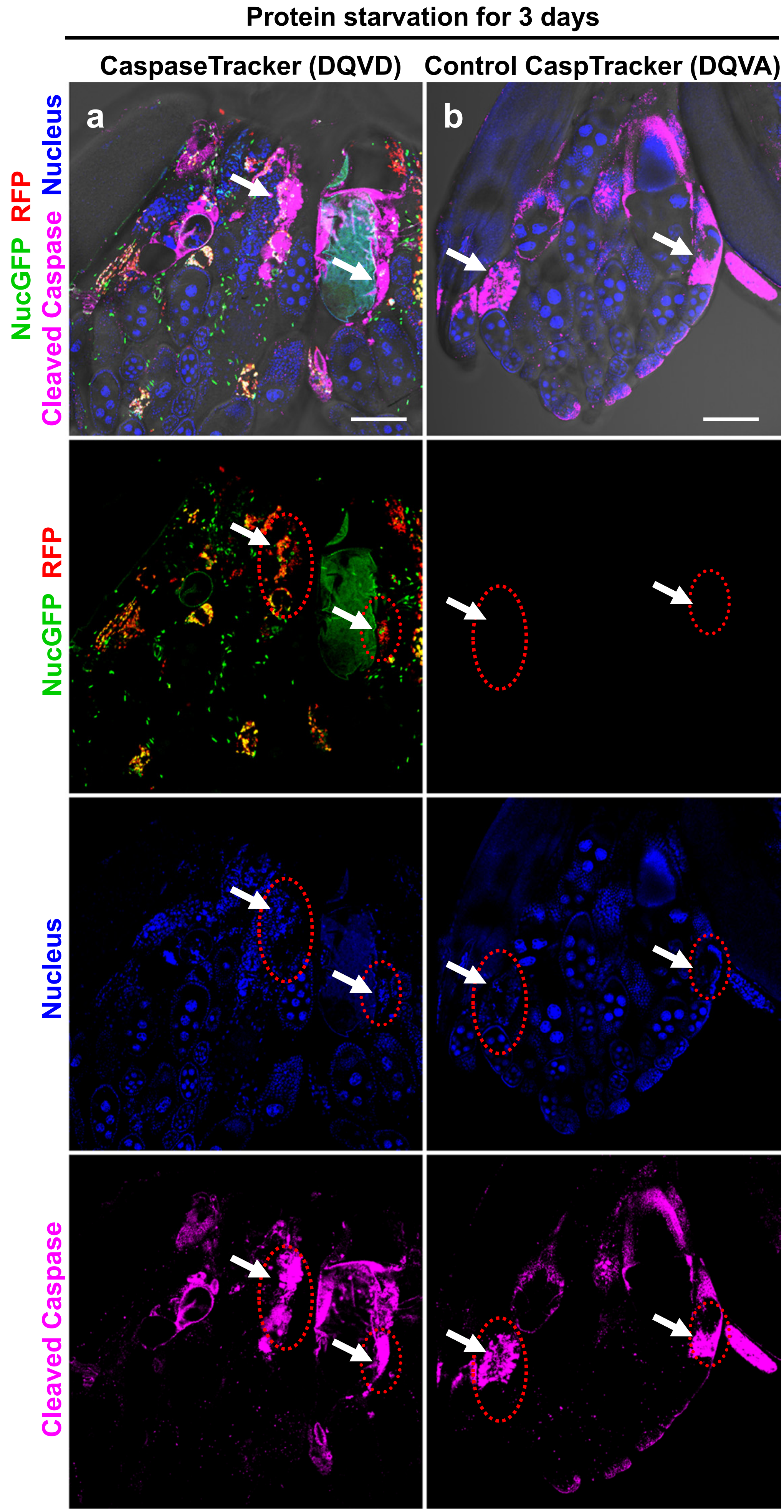

Protein starvation for 3 days
CaspaseTracker (DQVD)
Control CaspTracker (DQVA)
a
b
NucGFP RFP Cleaved Caspase Nucleus
NucGFP RFP
Nucleus
Cleaved Caspase

CaspaseTracker (DQVD), Recovery from Cold Shock

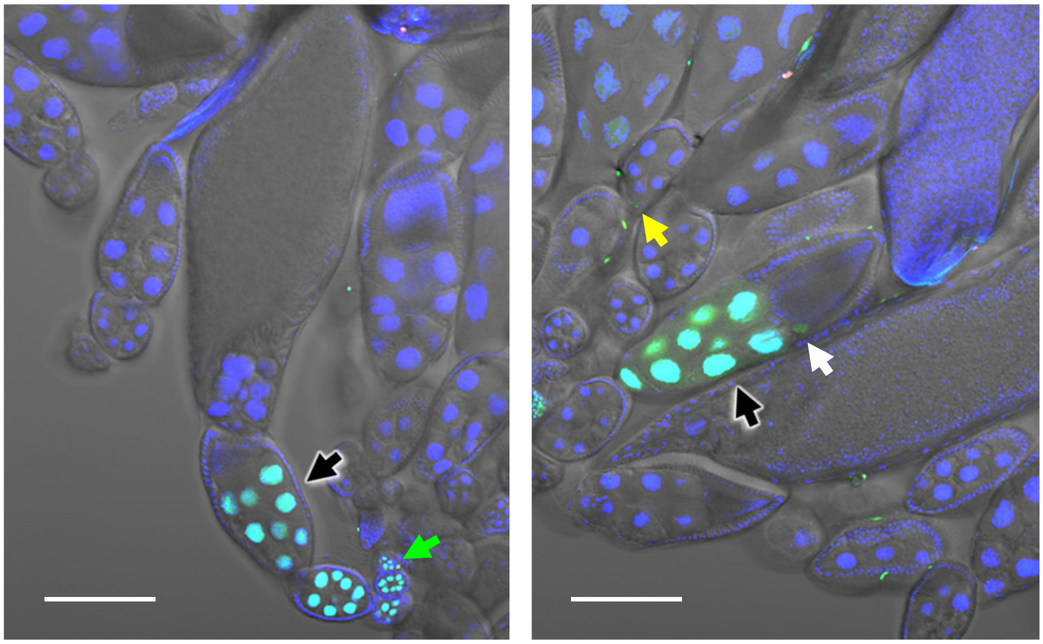

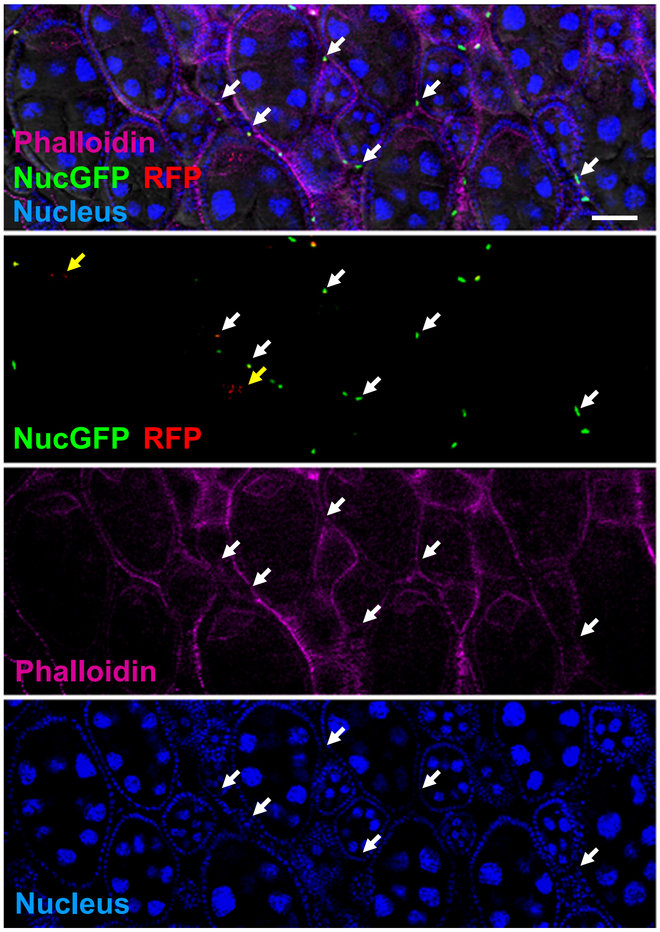
Phalloidin
NucGFP RFP
Nucleus
NucGFP RFP
Phalloidin
Nucleus

Tang et al. Supplementary Figure 6

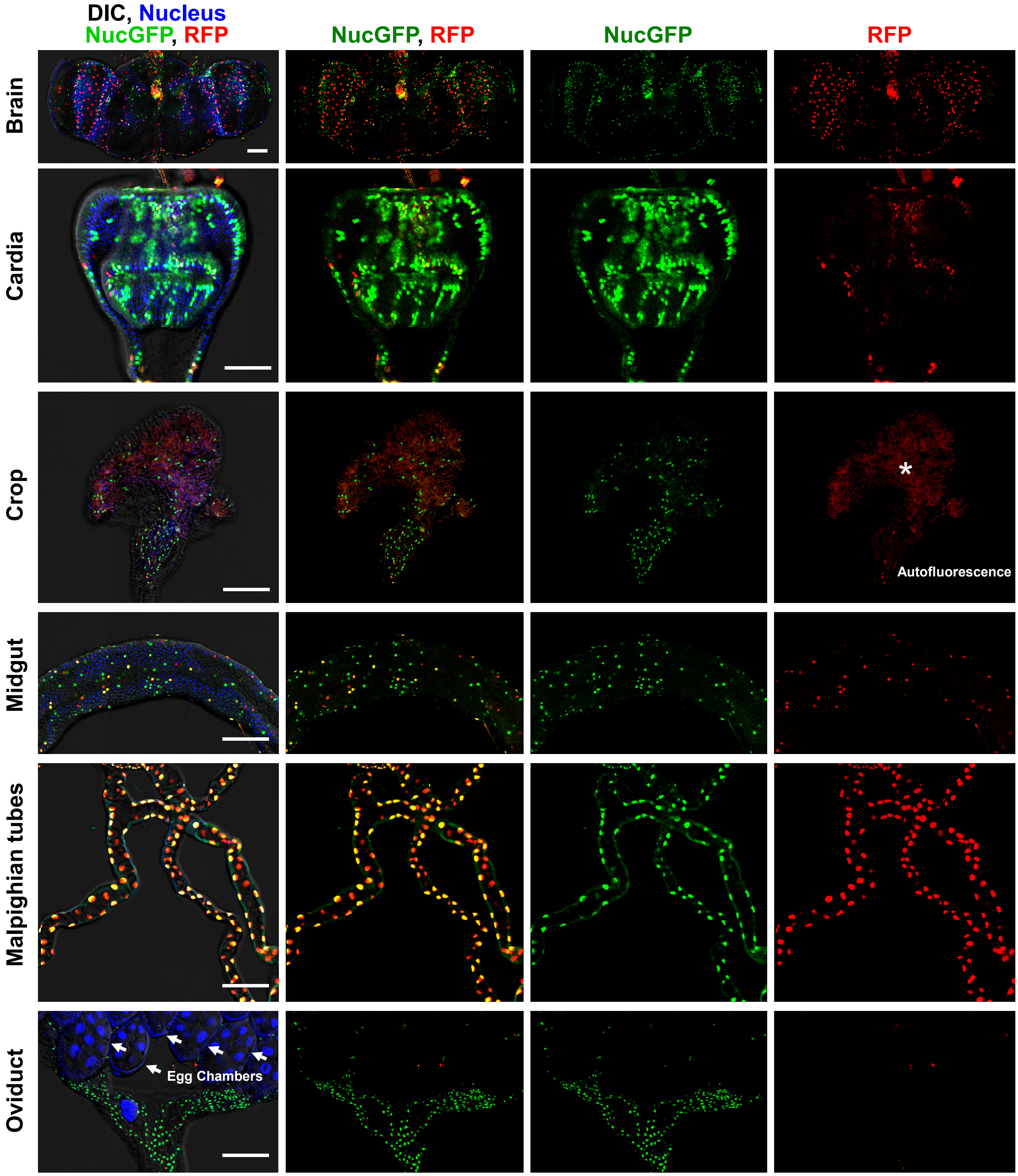

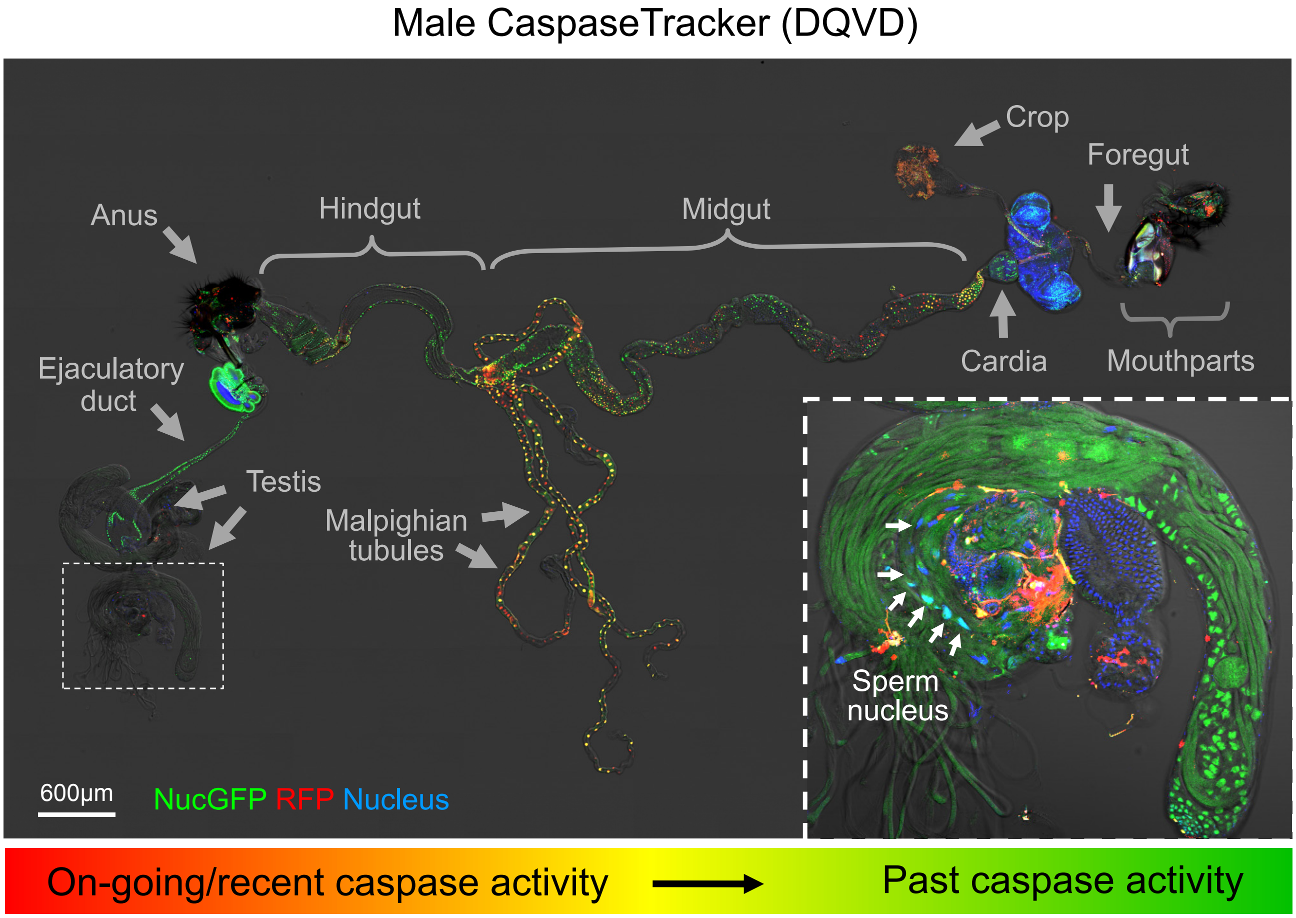
Male CaspaseTracker (DQVD)
Crop
Foregut
Anus
Hindgut
Midgut
Cardia
Mouthparts
Ejaculatory duct
Testis
Malpighian tubules
Sperm nucleus
600µm
NucGFP RFP Nucleus
On-going/recent caspase activity
Past caspase activity

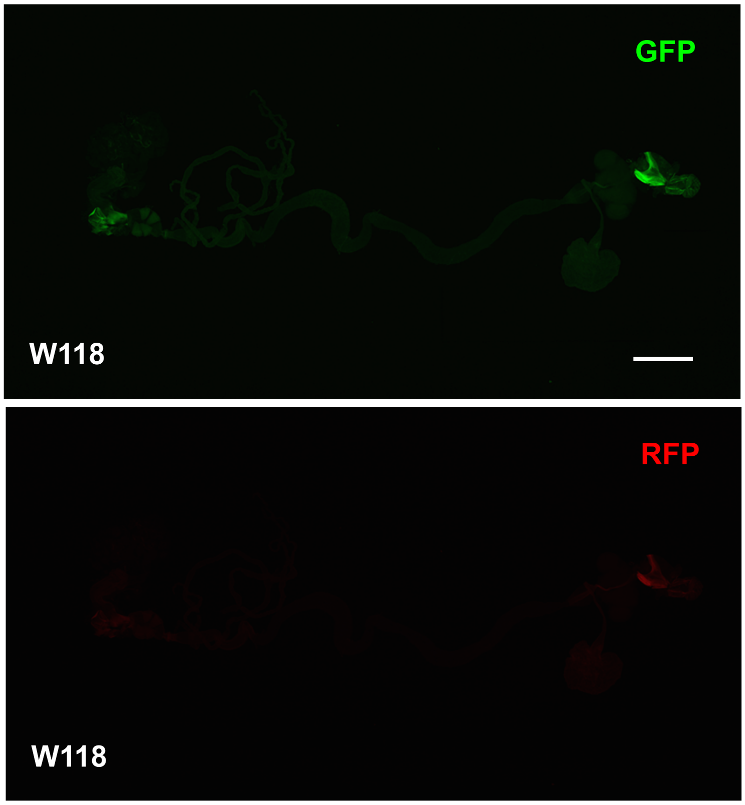
GFP
W118
RFP
W118

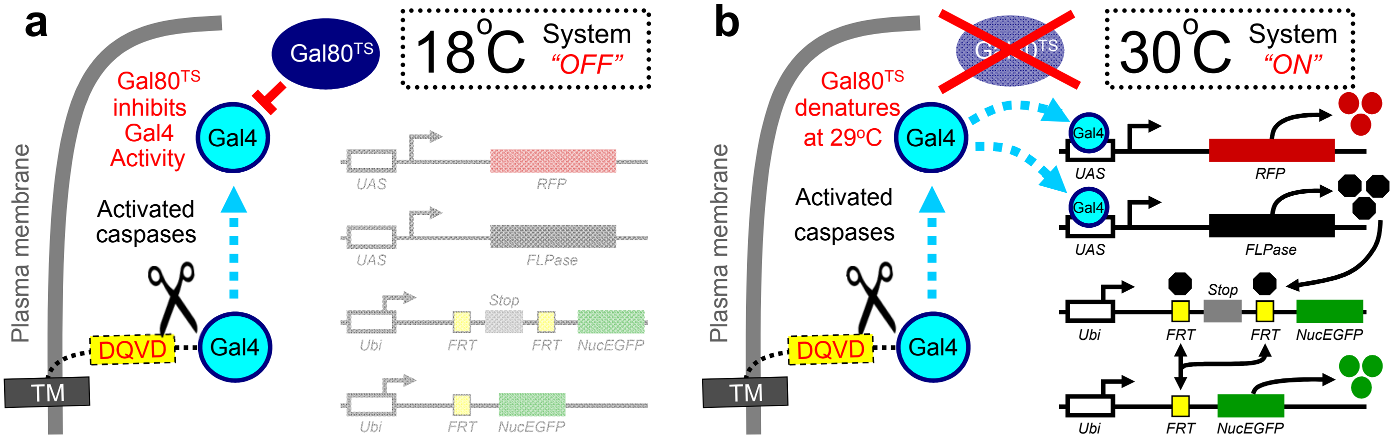
a
18°C System
"OFF"
Gal80TS
Gal80TS inhibits Gal4 Activity
Gal4
Activated caspases
Plasma membrane
DQVD
Gal4
TM
UAS
RFP
UAS
FLPase
Stop
Ubi
FRT
FRT
NucEGFP
Ubi
FRT
NucEGFP
b
30°C System
"ON"
Gal80TS denatures at 29°C
Gal4
Activated caspases
Plasma membrane
DQVD
Gal4
TM
Gal4
UAS
RFP
Gal4
UAS
FLPase
Stop
Ubi
FRT
FRT
NucEGFP
Ubi
FRT
NucEGFP
